# Successful Management of Infectious Crystalline Keratopathy with Intrastromal Antibiotic Injections

**DOI:** 10.1155/2022/5830617

**Published:** 2022-11-30

**Authors:** Luis Martinez-Velazquez, Kevin K. Ma, Neal S. Patel, Zhonghui Katie Luo

**Affiliations:** Massachusetts Eye and Ear Infirmary, 243 Charles St, Boston, MA 02114, USA

## Abstract

*Purpose.* To report the successful treatment of a case of presumed infectious crystalline keratopathy with repeated intrastromal antibiotic injections in a cornea graft in the setting of severe ocular graft-vs.-host-disease (GVHD). *Observations.* A 62-year-old man with a history of ocular GVHD and tectonic penetrating keratoplasty (PK) for corneal melt from herpes zoster keratopathy developed presumed infectious crystalline keratopathy (ICK) in the corneal graft. Given the patient's complicated ocular history, chronic immunosuppression and new cardiac comorbidities, a therapeutic PK would most likely fail. Efforts were then directed to rescue the graft with minimally invasive approaches. Two separate intrastromal injections of cefuroxime and moxifloxacin successfully treated his ICK. *Conclusions and Importance.* This case supports a role for repeated intrastromal antibiotic injections in patients with ICK refractory to topical antibiotic therapy, which might eliminate the need for therapeutic PK and preserve vision.

## 1. Introduction

Infectious crystalline keratopathy (ICK) is an indolent infective keratitis characterized by fine branch-like crystalline opacities in the anterior corneal stroma accompanied by minimal inflammation [1, 2]. Identification of the causative pathogens is often not possible given that cultures from corneal scrapings are often too superficial to reach the causative organisms, which are commonly located deep in the anterior stroma [2]. Gram-positive bacteria, in particular *Streptococcus mitis*, appear to be the most common causative organism among published reports of ICK with a positive corneal culture [2]. However, there have also been reports of ICK caused by gram-negative bacteria, fungus, or *Acanthamoeba* [3, 4].

ICK has been classically described in patients with topical immunosuppression following a penetrating keratoplasty (PK) and is notoriously difficult to treat [1, 5]. ICK has also been described in patients treated with immunosuppressive agents for other ocular or systemic conditions including Stevens-Johnson syndrome, and chronic graft-vs.-host disease (GVHD) [6, 7]. Although local or systemic immunosuppression often precedes ICK, this condition has been linked to neurotrophic corneal diseases such as herpetic keratitis, denervation after corneal surgery (PK [2, 8], laser-assisted *in situ* keratomileusis (LASIK) [9]), or even after long-term topical anesthetic use [10] in the absence of immunosuppression. Initial treatment usually consists of topical broad spectrum fortified antibiotics. These can then be refined based on microorganism sensitivities if a successful culture is obtained. Many cases require corneal transplantation if they are refractive to topical therapy.

Our case report describes the successful treatment of presumed ICK with two sequential sessions of intrastromal antibiotic injections in a patient that had previously undergone tectonic PK for corneal melt from herpes zoster keratopathy and ocular GVHD following allogeneic hematopoietic stem cell transplantation (allo-HSCT). This treatment modality successfully prevented progression of the infection and preserved his baseline best corrected visual acuity (BCVA) after twenty-three months from initial diagnosis, without requiring a repeat PK following failed topical antibiotics treatment.

## 2. Case Report

**Figure 1:**
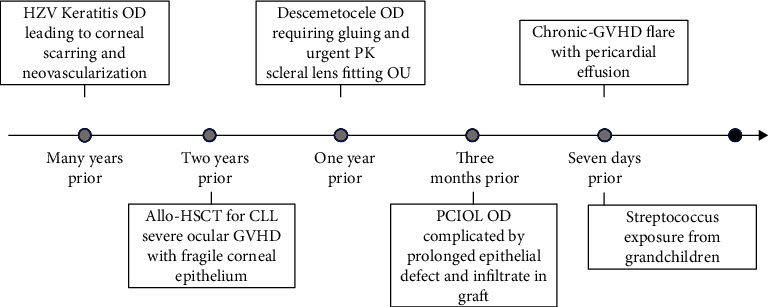
Timeline of history of present illness. Graphical representation of the ocular and systemic disease course for this patient.

A 62-year-old man with a complex ocular history presented to our ophthalmology clinic with blurry vision of the right eye for 2 days. His ophthalmic history was notable for herpes zoster virus (VZV) keratitis of the right eye, ocular GVHD in both eyes and a tectonic PK in his right eye PK following an urgent presentation for a descemetocele. Under the emergency circumstance of his initial presentation to our service, corneal sensation was not checked before his PK. He was fitted with scleral lenses (Boston Sight) in both eyes to improve his vision and support his fragile ocular surface.  Figure 1 outlines a timeline of his ophthalmic disease course. His chronic GVHD also involved multiple organ systems including his eyes, lungs, mouth mucosa, skin, liver, and soft tissues, which was classified as “moderate” based on the 2014 NIH consensus [11, 12].

Seven days prior to the onset of his blurry vision, he was hospitalized at an intensive care unit for pericardial effusion and new atrial fibrillation in the setting of a chronic systemic GVHD flare-up. Following his allo-HSCT, he had always required multiple systemic immunosuppression therapies for chronic GVHD management. After a recent hospital admission, his oral prednisone was increased from 10 mg daily to 50 mg daily. He was concurrently taking tacrolimus 2 mg daily, topical betamethasone (0.05%) ointment for his skin, undergoing extracorporeal photopheresis twice a week, and receiving monthly intravenous immunoglobulin (IVIG) infusions. His prophylactic treatment includes valacyclovir 1 g daily and trimethropim-sulfamethaxozole 400-80 mg daily. He was also on topical ocular immunosuppression including non-preserved prednisolone acetate 1% twice a day in the right eye and loteprednol 0.5% gel once a day in the left eye chronically in addition to frequent lubrication with nonpreserved artificial tears. His puncti were all occluded with silicone punctual plugs. He wore his scleral lenses daily in both eyes for 12 to 14 hours per day.

**Figure 2:**
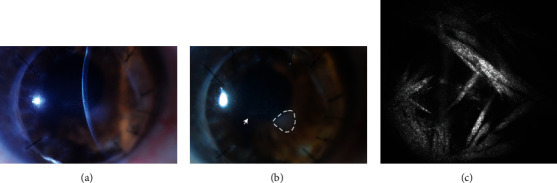
Slit-lamp photograph of the right eye at initial presentation. (a) A slit-beam photograph centered over the new crystalline pattern of branching thin structures in the anterior stroma. (b) A wide-beam photograph demonstrating the branch-like structures in the anterior stroma of the central visual axis (arrowhead), and nasally, a preexisting triangular anterior stromal scar (dashed outline). (c) Confocal microscopy revealed mid-stromal (195 *μ*m deep) highly reflective needle-like structures consistent with crystalline keratopathy, but not specific for an individual pathogen.

The ocular exam was notable for a decrease in vision in his scleral lens from 20/30 to 20/40, intraocular pressures of 14 mm Hg and rare cell in the anterior chamber in the right eye. His left eye had a stable vision in scleral lens of 20/25, intraocular pressure of 22 mm Hg with no new findings. Examination of the right cornea (Figure 2(a)) demonstrated a stable preexisting triangular 1.5 mm anterior stromal haze paracentrally and a new crystalline pattern of branching thin structures in the anterior stroma centrally, and keratic precipitates in the endothelium (Figure 2(b)). The left cornea was clear. Confocal microscopy revealed hyperreflective needle-like structures located in the mid stroma at approximately 195 *μ*m consistent with crystalline keratopathy, but nonspecific for an individual pathogen (Figure 2(c)). The central corneal epithelium was scraped and cultured, but unfortunately did not identify the causative organism. Fortified nonpreserved ceftazidime (50 mg/mL) and moxifloxacin (5 mg/mL) drops were started every hour as empiric therapy for gram-positive organisms. The likelihood of a gram-positive organism was reinforced when on further history; the patient reported that he was with his grandchild who had strep throat a few days prior to onset of symptoms (Figure 1).

One week after initial presentation, we found a stable appearance of the branch-like lesions, slight improvement of the epi-defect, and persistent anterior chamber inflammation despite the resolution of the keratic precipitates. To control the infection as early as possible, the patient was brought to the operating room for intrastromal and intracameral antibiotic injections. Given his systemic comorbidities, only topical lidocaine (3.5%) gel was used for anesthesia. Under the operating microscope, we injected 0.1 mL of 1 mg/0.1 mL nonpreserved cefuroxime intrastromally through a 30-gauge needle to the branch lesions, and 0.1 mL of 0.5 mg/0.1 mL moxifloxacin (Apotex pharmaceuticals) intracamerally using a 30-gauge irrigator. The moxifloxacin is commercially available and was aliquoted by our inpatient pharmacy under sterile conditions for use in the operating room. One hundred microliters of aqueous humor were collected from the anterior chamber for culture, which resulted negative for bacteria or fungi. The patient resumed wear of his scleral lens two days after the intrastromal injection to protect his ocular surface.

**Figure 3:**
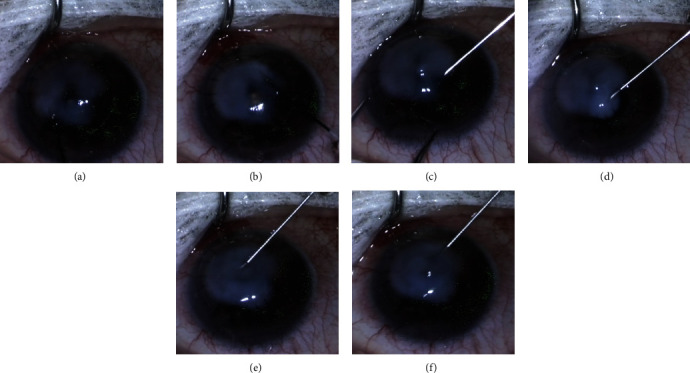
Image series from surgical video demonstrating the intrastromal antibiotic injections through a 30-gauge needle. The injections were made in a circular pattern surrounding the crystalline deposits, achieving a higher concentration centrally and keeping the needle tracks away from the visual axis (image progression from (a) to (f)). Full video available here in https://www.dropbox.com/s/x0gbutn8d64a5sd/Video1.mp4?dl=0.

Five weeks after the initial presentation and while on continued topical antibiotics, the patient reported subjectively worse vision, which was measured in his scleral lens at 20/30-2. On examination, the “branch-like” lesions were slightly larger in size and clearly invaded a deeper layer in the stroma with increased central stromal haze. While the first intrastromal injection of cefuroxime only transiently stalled but failed to control his ICK, other treatment options did not seem more promising at the time. Notably, the patient was not medically stable for surgery given his cardiac status with persistent atrial fibrillation, pericardial, and pleural effusion. Therefore, we administered a second round of intrastromal injections as previously described consisting of non-preserved 0.1 mL of 0.5 mg/0.1 mL moxifloxacin and 0.06 mL of 1 mg/0.1 mL cefuroxime injected through a 30-gauge needle in a circular pattern surrounding the “branches”. We settled on these volumes after reaching an appropriate degree of stromal edema, and with the intention to reduce the risk of a Descemet's membrane detachment. This sequence created a higher concentration centrally and kept the needle tracks away from the visual axis (Figure 3). We discontinued topical antimicrobials following the procedure, and two days later, the patient resumed his scleral lens wear in the right eye.

**Figure 4:**
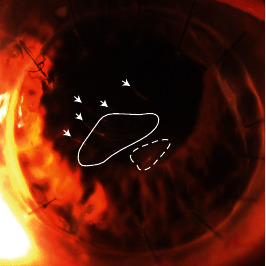
Slit-lamp photograph of the right eye 23 months after initial presentation. The “branch-like” infiltrates were still visible centrally (solid outline). Multiple linear needle tracks from the intrastromal injections were in the paracentral cornea stroma (arrowheads). The triangular 1.5 mm anterior stromal haze was stable in appearance (dashed outline).

Five weeks after the second round of intrastromal injections, the patient reported subjective improvement in his right eye vision, which was measured in the same scleral lens at 20/25, despite the residual needle tracks adjacent to his visual axis. Twenty-three months after his intrastromal injections, he continues doing well with a stable vision of 20/25 in the right eye while wearing his current scleral lens. The appearance of the branch like structures was mostly stable with slightly less defined borders without any further increase in size (Figure 4). His left eye did stably well throughout the whole disease course.

## 3. Discussion

ICK is an indolent infectious keratitis characterized by fine branch-like crystalline opacities commonly found in the anterior stroma [1, 2]. Initial inoculation occurs through an epithelial defect, and the microorganisms are then able to proliferate through the space between the lamellar planes of the stroma and the crisscrossing cell bodies of the keratocytes. Immunosuppression enables the crystalline appearance of this condition by suppressing the inflammatory reaction and infiltration of immune cells and edema seen in other types of infectious keratitis.

Cases of ICK may be asymptomatic or present with some of the symptoms of keratitis including decreased visual acuity, photophobia, and pain. The degree of conjunctival injection and adjacent inflammation is usually lower than in other forms of keratitis due to the indolent nature of the infection and the common association with long-term use of topical corticosteroids [13].

Our patient was immunosuppressed through multiple mechanisms including systemic and topical steroids to address complications of his GVHD secondary to his allo-HSCT. Posttransplantation patients suffer from severe immune deficiency due to thymic damage, and secondary to pharmacologic immunosuppressive agents used for GVHD prophylaxis [14]. Ocular GVHD affects more than 50% of these patients reducing their quality of life significantly through ocular manifestations ranging from irritation, poor vision to nonhealing epithelial defects, corneal melt, intractable pain, and irreversible vision loss [15]. Treatment for moderate to severe chronic GVHD in general requires systemic and topical immunosuppression such as steroids and calcineurin inhibitors, which increases the risk of infections and impairs healing.

ICK has also been previously linked to neurotrophic corneal diseases such as herpetic keratitis, denervation after corneal surgery (PK [2, 8], laser-assisted *in situ* keratomileusis (LASIK) [9]), or even after long-term topical anesthetic use [10] in the absence of immunosuppression. Neurotrophic conditions are associated with delays in corneal wound healing [16] which provide an opportunity for the initial seeding of the cornea by a pathogen. Our patient initially presented to our care in the setting of a descemetocele requiring urgent PK; therefore, as previously mentioned corneal sensation was not checked prior to the PK or subsequently after the PK. Nevertheless, a degree of neurotrophic keratitis is presumed given expected denervation from the PK and his prior history of herpetic keratitis.

Many of the microbial species that cause ICK form biofilms, which limit the effectiveness of topical antimicrobials. Meisler and colleagues reported a case of ICK in which they cultured *Streptococcus viridans* and measured a minimum inhibitory concentration for antibiotic treatment four times greater than what was required to treat this species in other contexts [5]. It was later proposed that this was due to the protective effects of a biofilm [2]. Historically, PK is often required to remove the infected corneal tissue, or to restore vision in cases with severe corneal scarring despite successful treatment of the infection. However, our patient's unique medical and ocular history rendered him a poor candidate for repeat PK.

Intrastromal injections of antibiotics have been suggested to treat ICK refractory to topical antimicrobials. Khan and colleagues reported successfully treating ICK caused by *Streptococcus mitis* and *Streptococcus parasanguinis* with intrastromal injection of cefuroxime [17]. There have also been reports of successful intrastromal amphotericin B and voriconazole to treat fungal keratitis [18, 19]. Injections into the anterior stroma have the advantage of delivering higher concentrations of antimicrobials directly where the microorganisms are located and overcome the barrier set by biofilms. While we considered injecting voriconazole due to its effectiveness for treating fungal cases, we decided to focus initial therapy on agents that would cover gram positive organisms given that they are the most frequent cause of crystalline keratopathy. This focus was important to minimize the total volume of antimicrobials injected to the stroma to avoid possible complications such a Descemet's membrane detachment.

Corneal biopsies have been used with success to identify the causative microbe in atypical cases of crystalline keratopathy [20]. This approach should be given fair consideration when adjusting the antimicrobial therapy in a treatment refractory case. Our patient, however, had a deep (~195 *μ*m per confocal) and central lesion. A corneal biopsy would have certainly caused a large epithelial defect and risk delayed healing or scarring. The decision to proceed with empiric antimicrobial therapy was based on maximizing the chances of saving the corneal graft given that he was a poor surgical candidate for a PK.

Our case report is unique in that it demonstrates therapeutic success of treatment of ICK by sequential intrastromal antibiotic injections in an immunocompromised patient with ocular GVHD. The first intrastromal injection of 0.1 mL of 1 mg/0.1 mL cefuroxime only stalled the progression of ICK for a few weeks, showcasing the role of biofilms in antimicrobial treatment resistance. The second intrastromal injection of 0.1 mL of 0.5 mg/0.1 mL moxifloxacin and 0.06 mL of 1 mg/0.1 mL cefuroxime delivered a high concentration of two antibiotics simultaneously, which likely reached the bactericidal level and eradicated the organism. After the second intrastromal injection, the patient did not require additional antimicrobial therapies and has maintained good visual acuity at the most recent 2-year follow-up. The interbranch spaces remained clear without any further increase in the number or size of the branches for two years, indicating that the injections were an effective treatment.

There are no current guidelines for the selection of antibiotic agents, concentration, or injection volume for such unconventional treatment. We chose the nonpreserved preparations of moxifloxacin and cefuroxime, at higher concentrations than previously reported [8]. The concentrations were chosen because they have been routinely used for intracameral injection in cataract surgeries world-wide [21]. We limited the delivered volume of the antibiotics (only 0.06 mL of cefuroxime was injected) to avoid a Descemet's membrane detachment from the significant stromal edema created by the injections. We also aimed to place our needle-tracks outside of the visual-axis. Within one to two days after each intrastromal injection, the patient was able to resume wear of his scleral lens, which provided him with essential surface protection and sharper vision. Merely two weeks after the second injection, the patient regained 20/40 vision in his scleral lens, which would have been impossible had he undergone a repeat PK. To date, more than two years after the intrastromal injections, there were no signs of endothelial dysfunction or other signs of corneal toxicity. Our case indicated the more concentrated stromal injections were well tolerated by the endothelium of a corneal graft. The prominence of the needle-tracks has reduced with time, and while they are not affecting his vision, they are still visible on examination.

## 4. Conclusions

This novel strategy expands our treatment options for ICK intractable to topical antibiotics. We propose that this treatment can be considered before a therapeutic PK and/or in patients with high risk of graft failure. Future controlled trials are needed to determine the treatment efficacy of this approach in comparison to therapeutic PK.

## Data Availability

The relevant data for this case is included in the case report.
